# Preface: Experiencing Cancer in Appalachian Kentucky

**DOI:** 10.13023/jah.0203.08

**Published:** 2020-07-19

**Authors:** P.Michele Ellison, Robin C. Vanderpool

**Affiliations:** Chair, Connect2HealthFCC Taskforce, Deputy General Counsel, Federal Communications Commission; Chief, Health Communication and Informatics Research Branch, National Cancer Institute

**Keywords:** Appalachia, cancer care, broadband, communications, connectivity, rural health

## Abstract

Connected cancer care is of increasing importance in light of the COVID-19 pandemic. The Linking & Amplifying User-Centered Networks through Connected Health (L.A.U.N.C.H.) Collaborative in Appalachian Kentucky has pioneered a new roadmap for equipping communities with the transformative power of broadband to innovate around the future of cancer care and to better scale their ideas. The roadmap involves reaching across disciplines, including public health, anthropology, telecommunications, and user-centered design. The goal is to leverage connectivity and cancer communication research and practice to make a real difference for patients and families.

Connected cancer care delivered remotely and effectively to rural and underserved communities has been a long sought-after goal given the well-recognized disparities in cancer incidence and mortality across geographies.^1^ However, the COVID-19 pandemic has rapidly transformed this unmet need and unrealized opportunity into a public health imperative, both within and beyond rural America. In a June 2020 editorial in *Science*, Dr. Ned Sharpless, Director of the National Cancer Institute, emphasized that the COVID-19 pandemic is causing “delayed diagnosis and suboptimal care for people with cancer” and could lead to almost 10,000 excess deaths from breast and colorectal cancer alone.^2^

It is against this backdrop—of both existing and emerging public health challenges—that the Linking & Amplifying User-Centered Networks through Connected Health (L.A.U.N.C.H.) Collaborative’s work in Appalachian Kentucky —at the intersection of broadband and cancer—becomes unusually prescient. Notably, we have seen significant progress on evidence-based, connected cancer care solutions in the last few years, making ubiquitous connectivity particularly important for more widespread and large-scale technology diffusion. For instance, telehealth applications successfully facilitate digital “conversations” between cancer patients and healthcare providers, and other connected cancer care solutions focus on smoking cessation, early detection, genetic counseling, or remote symptom monitoring and management—allowing providers to remotely and rapidly intervene in a patient’s care.

At the same time and despite overall gains, the “digital divide” persists in parts of rural America, putting connected health applications out of reach for many cancer patients. For example, in 2018, Internet adoption was 48.7% in Kentucky compared to a national average of 67%.^3^ According to the Pew Research Center, rural Americans also report lower levels of technology adoption. Roughly three-in-ten rural adults do not own a smartphone (29%) or desktop computer (31%), and 51% report not having a tablet computer.^4^

The National Cancer Institute and Federal Communications Commission, as lead federal agency partners in the L.A.U.N.C.H. Collaborative,^5^ recognize that policymakers and communities must leverage every tool at our disposal to address these connectivity and cancer care challenges. The need to ensure that cancer interventions are patient-centered—and the reality that a perceived lack of relevance is one key driver of non-adoption of broadband^6^—required a new approach. We sought a deeper understanding of the historical and cultural crosscurrents in various communities of interest that could influence the effectiveness of our interventions and policy approaches. We reached across disciplines, including public health, anthropology, telecommunications, and user-centered design, and commissioned various studies, including the ethnographic review focused on Appalachian Kentucky, in the following article.^7^ Our ambitious goal is to bridge community-driven design, broadband connectivity, and connected health technology to improve cancer outcomes and quality of life for patients in rural and historically underserved areas.^8^ Specifically, we considered the scholarship of various researchers, including two anthropologists who offer critiques of the typical “development narrative” as a window into the broader context that the L.A.U.N.C.H. initiative might confront.

The study described in the next article^7^ was also intended to place a wide range of local perspectives front and center. We take this opportunity to extend our gratitude to the local stakeholders who candidly shared their insights and experiences, including cancer patients, caregivers, healthcare professionals, and technology providers. This new roadmap—for equipping communities with the transformative power of broadband to innovate around the future of cancer care and to better scale their ideas—would not have been possible without their generosity; nor would we have been able to shed critical light on the availability of virtual avenues for COVID-19 response and related public health interventions.

We came to this project with diverse backgrounds, perspectives, and areas of expertise, the value of which cannot be understated. Although our nomenclature and analytical lenses may vary and we may express ourselves in different terms, we speak with one voice on the importance of leveraging connectivity and cancer communication research and practice to make a real difference in the health and well-being of Appalachians, and indeed all Americans. In these extraordinary times, it is both a policy and moral imperative.
